# Combining the Estimated Date of HIV Infection with a Phylogenetic Cluster Study to Better Understand HIV Spread: Application in a Paris Neighbourhood

**DOI:** 10.1371/journal.pone.0135367

**Published:** 2015-08-12

**Authors:** Olivier Robineau, Pierre Frange, Francis Barin, Françoise Cazein, Pierre-Marie Girard, Marie-Laure Chaix, Georges Kreplak, Pierre-Yves Boelle, Laurence Morand-Joubert

**Affiliations:** 1 Service Universitaire Régional de Maladies Infectieuses et Tropicales, Centre Hospitalier de Tourcoing, Tourcoing, France; 2 INSERM, UMR_S 1136, Institut Pierre Louis d’Epidémiologie et de Santé Publique, Paris, France; 3 EA 3620, Université Paris Descartes, Sorbonne Paris-Cité, Paris, France; 4 Laboratoire de Microbiologie & Unité d’Immunologie, Hématologie et Rhumatologie Pédiatriques, AP-HP, Hôpital Necker, Enfants Malades, Paris, France; 5 Centre National de Référence du VIH & INSERM U966, CHU Bretonneau & Université Francois Rabelais, Tours, France; 6 Institut de Veille Sanitaire, Saint-Maurice, France; 7 Service des Maladies Infectieuses et Tropicales, Hôpital Saint Antoine, APHP, Paris, France; 8 Laboratoire de Virologie, AP-HP, Hôpital Saint-Louis, Paris, France; 9 Laboratoire du Chemin Vert, Paris, France; 10 Sorbonne Universités, UPMC Univ Paris 06, UMR_S 943, Paris, France; 11 Service de Santé Publique, AP-HP, Hôpital Saint-Antoine, Paris, France; 12 INSERM, UMR_S 943, Paris, France; 13 Laboratoire de Virologie, Hôpital Saint-Antoine, AP-HP, Paris, France; Institut Pasteur of Shanghai,Chinese Academy of Sciences, CHINA

## Abstract

**Objectives:**

To relate socio-demographic and virological information to phylogenetic clustering in HIV infected patients in a limited geographical area and to evaluate the role of recently infected individuals in the spread of HIV.

**Methods:**

HIV-1 *pol* sequences from newly diagnosed and treatment-naive patients receiving follow-up between 2008 and 2011 by physicians belonging to a health network in Paris were used to build a phylogenetic tree using neighbour-joining analysis. Time since infection was estimated by immunoassay to define recently infected patients (very early infected presenters, VEP). Data on socio-demographic, clinical and biological features in clustered and non-clustered patients were compared. Chains of infection structure was also analysed.

**Results:**

547 patients were included, 49 chains of infection containing 108 (20%) patients were identified by phylogenetic analysis. analysis. Eighty individuals formed pairs and 28 individuals were belonging to larger clusters. The median time between two successive HIV diagnoses in the same chain of infection was 248 days [CI = 176–320]. 34.7% of individuals were considered as VEP, and 27% of them were included in chains of infection. Multivariable analysis showed that belonging to a cluster was more frequent in VEP and those under 30 years old (OR: 3.65, 95 CI 1.49–8.95, p = 0.005 and OR: 2.42, 95% CI 1.05–5.85, p = 0.04 respectively). The prevalence of drug resistance was not associated with belonging to a pair or a cluster. Within chains, VEP were not grouped together more than chance predicted (p = 0.97).

**Conclusions:**

Most newly diagnosed patients did not belong to a chain of infection, confirming the importance of undiagnosed or untreated HIV infected individuals in transmission. Furthermore, clusters involving both recently infected individuals and longstanding infected individuals support a substantial role in transmission of the latter before diagnosis.

## Introduction

In France, an estimated 150,000 persons are currently living with HIV, and 7000 new infections occur each year. As in many Northern countries, HIV incidence remains high among men who have sex with men (MSM) [1]. The respective role of recently infected patients or chronically infected patients with undiagnosed or untreated HIV infection in the spread of the epidemic has been discussed [2,3]. It remains a key concern for the design of prevention policies such as pre-exposure prophylaxis or treatment as prevention (PrEP and TasP). Phylogenies of highly genetically variable viruses such as HIV-1 are potentially informative when dissecting the dynamics of the epidemic [4,5]. Several studies have demonstrated the presence of clusters of highly related HIV-1 sequences, particularly among HIV-positive individuals with recent infection [6–8]. Modelling studies based on a phylogenetic or behavioural approach have estimated that between 20% and 90% of onward transmissions results from undiagnosed patients in the MSM population [9,10]. Depending on the models, recent infections are involved in 4% to 50% of onward transmissions [3,6,8,10,11,12]. Our study had the following objectives: to assess the benefit of associating virological data to phylogenetic analysis in a restricted geographical area with particular epidemiological characteristics and to evaluate the role of recently infected individuals in the spread of HIV.

For this, we combined phylogenetic analysis based on available HIV sequences obtained from genotypic resistance testing and the use of an enzyme immunoassay (EIA-RI) to identify recently infected patients. This study took place in a population of treatment-naïve patients receiving follow-up in an area of Paris (Le Marais) that is home to a large MSM community and where both prevalence and incidence of HIV-1 are high in the MSM population.

## Patients and Methods

### Ethics

The study was approved by the French authorities (Comité consultatif sur le traitement de l’information en matière de recherche dans le domaine de la santé (CCTIRS) and Commission nationale de l’informatique et des libertés (CNIL)). Patients gave their written informed consent for the use of their clinical and biological data collected during their medical follow-up for the purpose of retrospective study. This procedure was approved by CCTIRS and CNIL.

### Study population

Since 2008, French national guidelines recommend a genotypic resistance test (GRT) be performed at the time of diagnosis and/or before initiation of Highly active antiretroviral therapy (HAART) [13]. The study included any newly diagnosed and/or treatment-naive patient who had a blood sample for a GRT between 2008 and 2011 in two laboratories (Le Chemin Vert, a private laboratory and the virological laboratory of Saint-Antoine Hospital, Paris). These two laboratories cover most of the HIV GRTs performed in an area of Paris overlapping the 3rd, 4th and 12th districts where the gay community is highly represented (Le Marais). Patients received follow-up from physicians belonging to a network dedicated to HIV care. Epidemiological and clinico-biological data, such as age, mode of transmission, ethnic origin, date of diagnosis, date of treatment initiation, viral load and CD4 T-cell count at time of GRT, were collected by using standardized questionnaires filled out by the attending physician and the laboratory where the analyses were performed. The epidemiological, clinical and therapeutic data were collected in March 2013.

### Genotypic resistance analysis

GRTs were performed in the virological laboratory of Saint-Antoine Hospital. Drug resistance was evaluated by amplifying and sequencing the HIV-1 reverse transcriptase (RT), protease and integrase genes from plasma samples by using the consensus technique of the ANRS Resistance Study Group (http://www.hivfrenchresistance.org), or the ViroSeq sequencing-based HIV-1 genotyping kit (Abbott, Rungis, France). Protease and reverse transcriptase mutations were identified from the consensus statement of the list for genotypic surveillance of the transmission of drug-resistant HIV-1 variants [14]. Resistance to nucleoside reverse transcriptase inhibitors (NRTI), non-NRTI (NNRTI), protease inhibitors (PI) and integrase inhibitors (IN) was defined according to the 2011 ANRS HIV-1 genotypic resistance interpretation algorithm [15].

### Phylogenetic analysis

The RT nucleotide sequences were aligned with previously reported representatives of group-M subtypes and circulating recombinant forms (CRFs) for which sequences are available in the HIV database (http://hiv-web.lanl.gov), using Clustal W (v1.7) with minor manual adjustments [16]. Nucleotide positions where resistance mutations are documented were excluded for tree inference. Phylogenetic interrelationships among viral RT sequences were estimated by using Neighbour-joining (NJ) [17] and maximum likelihood (ML) [18], using MEGA4 [19] and phyML (under the Hasegawa-Kishino-Yang (HKY85) model of evolution with a ratio a transversion: transition of 2: 1) [20], respectively. Bootstrap analysis with 1000 replicates was performed on the reconstructed phylogenetic trees. The NJ and ML methods produced very similar trees (data not shown). Chains of infection were defined as a clade of patients infected with strains whose phylogenetic analysis revealed a strong homology, suggesting very short or direct transmission chain of the virus. Clusters were defined as those clades with a support bootstrap value of 980/1000 or more and short branch lengths (genetic distances <0.015), as previously described [21]. Sequences located within clusters were validated for congruent polymorphisms and mutational motifs. We defined chains of infection of more than 2 individuals as “clusters” and the chains of 2 individuals as “pairs”

### Estimation of duration of infection

Frozen plasma from patients included in this study were used to discriminate between recent or chronic infections using an enzyme immuno-assay (EIA-RI) able to identify infections acquired less than six months before blood collection. In this assay, results are obtained through an algorithm that combines standardized measures of antibody binding to the immunodominant epitope (IDE) of gp41 and the V3 region of gp120 [22]. The quantitative outcome is the p-value of the algorithm, which increases with time since onset of the infection and for which a cut-off value of 0.5 was defined in order to optimally discriminate recent (less than six months) from long-standing infections. Specimens that registered less than or equal to 0.5 were considered to be recent infections, whereas those that registered over the 0.5 threshold were considered to be longstanding infections [22].

Patients with an EIA-RI suggestive of acquired infection less than six months before the test were defined as very early presenters (VEP). Among other patients, a sub-classification was performed based on the CD4 cell count. It included early presenters (EPs), late presenters (LPs) and extremely late presenters (ELPs), identified as having a CD4 cell count above 500/mm^3^, between 500/mm^3^ and 200/mm^3^ and below 200/mm^3^, respectively.

### Statistics

Comparisons between clustered and non-clustered populations were made using the chi-square or the Fisher’s exact test for categorical variables and the t-test or the Wilcoxon test for continuous variables. A descriptive analysis of the population was also performed. Among the subpopulation for which the EIA-RI was available, a logistic regression was performed to study the factors independently associated with belonging to a chains of infection, a pair or a cluster. To determine if the distribution of VEP into chains of infections could be due to chance, we compared the observed proportions of clusters containing only VEP, only non-VEP and a mixture of both with expected proportions if distribution into clusters had occurred randomly. More precisely, we permuted the individuals included into chains 1000 times and recorded the proportion of the three different chains’ structures of interest cited above. Comparison of the observed and theoretical distribution was done using a chi-square test. The statistical analysis was done by using R.15.0 [23].

## Results

From 2008 to 2011, 547 individuals with available GRTs were included in the study. Sexual intercourse among MSM was the most commonly reported route of transmission (n = 438, 92%). Heterosexual intercourse (n = 35, 7%), intravenous drug use (n = 1, 0.5%) and one mother-to-child infection (n = 1, 0.5%) were reported for other patients. Most individuals were born in France (n = 365, 76.5%) and reported having been infected in France (n = 320, 75.9%). The median age at infection was 36 years old (Inter Quartile Range (IQR): 31–42.75). GRT was done at time of diagnosis in 71% of the cases. The median viral load and CD4 rate at time of GRT were 4.69 log copies/ml (IQR: 4.25–6.00) and 447/mm^3^ (IQR: 338–616), respectively. Ten percent of the population was infected by HIV-1 variants harbouring resistance-associated mutations for at least one class of antiretroviral drugs. The CDC clinical stage at time of the genotype was A in 92.5%, B in 5.2% and C in 2.3% of the cases, while 77.6% of the viruses belonged to subtype B. In the subpopulation whose GRT was performed during follow-up (29%), the median time from diagnosis was three years (IQR: 2 to 5). Table 1 shows the baseline characteristics of the patients.

**Table 1:** Comparison of the characteristics of chained and non-chained population.

	Patient (nb)	Total population (CI)	Non-chains	Chains	p
Age at time of genotype	547	36 (31–42.8)	37	34	0.001
Transmission group (MSM) (%)	475	92.2 (89.3–94.4)	91.6	94.8	0.40
Country of infection (France) (%)	522	75.9 (71.9–79.4)	74.8	80	0.33
Country of birth (%)	477	76.5 (72.4–80.2)	77.4	72.9	0.43
Genotype performed at time of diagnosis or after (%)	515	71.1 (66.9–74.9)	67.5	85.9	0.00
Subtype B (%)	547	77.3 (80.7–77.9)	77.9	75	0.60
Viral load (log_10_)	547	4.7 (4.3–6)	4.7	4.8	0.20
CD4 (mm^3^)	526	447.5 (338.3–615.5)	441	479	0.07
**Time lapse between infection and genotyping**	449				
VEP (%)	156	34.8 (30.4–39.4)	31.8	46.7	0.02
EP (%)	101	22.5 (18.8–26.7)	23.4	18.9	0.47
LP (%)	164	36.5 (32.1–41.2)	37.6	32.2	0.46
ELP (%)	28	6.2 (4.3–9)	7.2	2.2	0.14
**Resistance to HAART**					
Resistance to at least one INTI (%)	547	4.4 (2.9–6.6)	4.6	3.7	0.90
Resistance to at least one INNTI (%)	547	5.7 (3.9–8)	5.9	4.6	0.77
Resistance to at least one PI (%)	547	3.1 (1.9–5)	3.2	2.8	0.93
Resistance to at least one HAART (%)	547	10.1 (7.7–13)	10.7	7.4	0.40

nb: number; CI: confidence interval; MSM: men who have sex with men. VEP: very early presenter; EP: early presenter; LP: late presenter; and ELP: extremely late presenter.

Dating infection by the EIA-RI was attempted in 461 individuals whose sera were still available. CD4 T-cell count was available for 449 of them. Based on the EIA-RI assay and CD4 T-cell count, 34.7% were defined as VEP, 22.5% were defined as EP, 36.5% as LP and 6.2% as ELP. CD4 T-cell count was below 500/mm^3^ for 46.2% of VEP.

### Characteristics of paired, clustered and non-chained population

**Fig 1:**
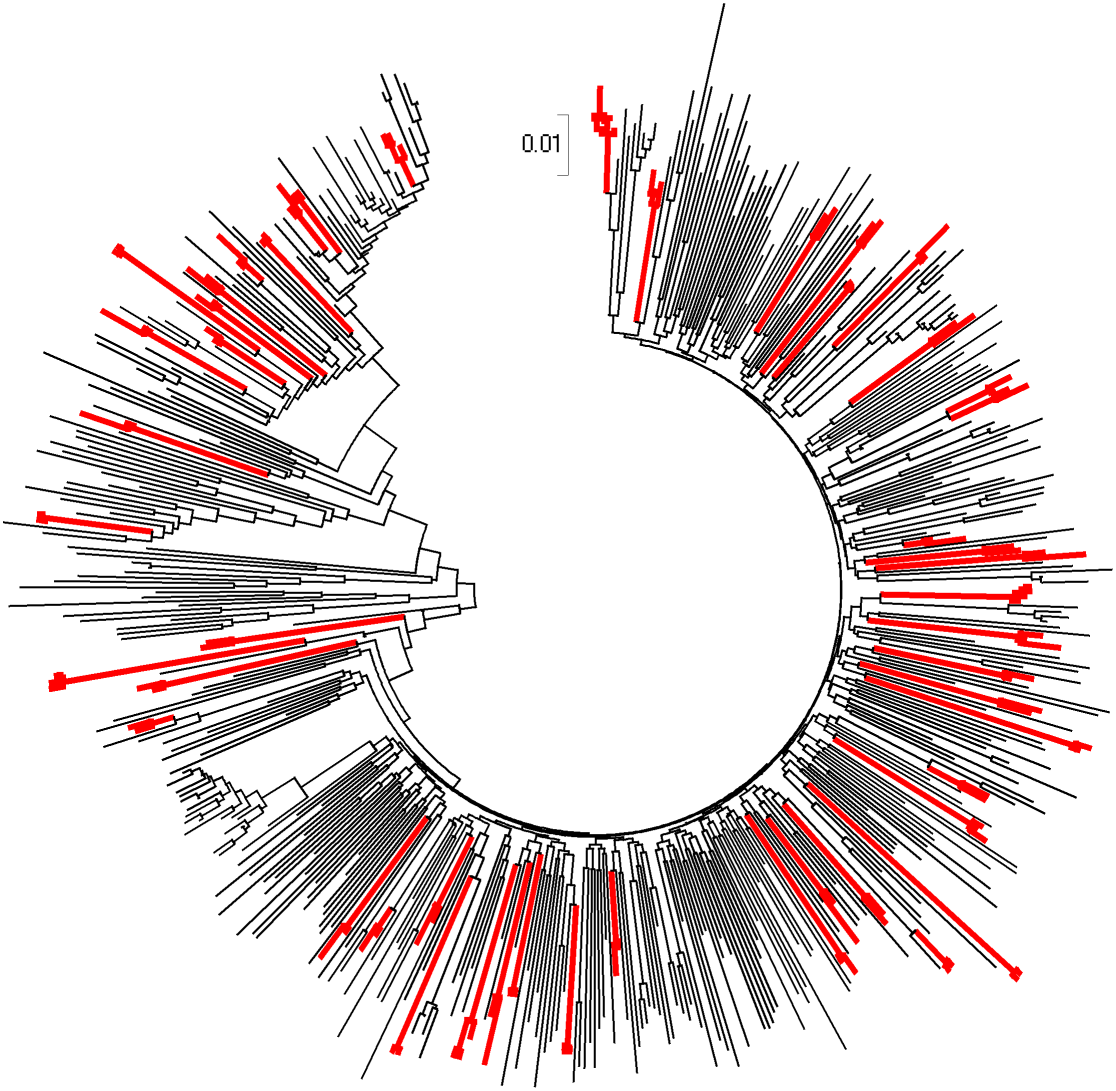
Phylogenetic tree of the entire population. The evolutionary history was inferred using the Neighbour-Joining method [17]. The optimal tree with the sum of branch length = 11.53297849 is shown. The tree is drawn to scale, with branch lengths in the same units as those of the evolutionary distances used to infer the phylogenetic tree. The evolutionary distances were computed using the Maximum Composite Likelihood method and are in the units of the number of base substitutions per site. All positions containing alignment gaps and missing data were eliminated only in pairwise sequence comparisons. There were a total of 1308 positions in the final dataset. Branches corresponding to clusters are in red.

Forty-nine chains of infection containing 19.7% (108/547) patients were identified. Fig 1 shows the phylogenetic tree of the entire population. Twenty-height of these individuals were involved in clusters and 80 in pairs. Median age at time of genotyping was statistically different between non-chained and paired or clustered population (37 vs 34 years old, p<0.001). The proportion of drug resistant variant in the paired or clustered and non-chained population was similar (7.4% vs 10.7%, p = 0.4) (Table 1). However, one pair contained two variants harbouring multidrug resistance (MDR) to RT-, protease- and integrase-inhibitors classes.

**Fig 2:**
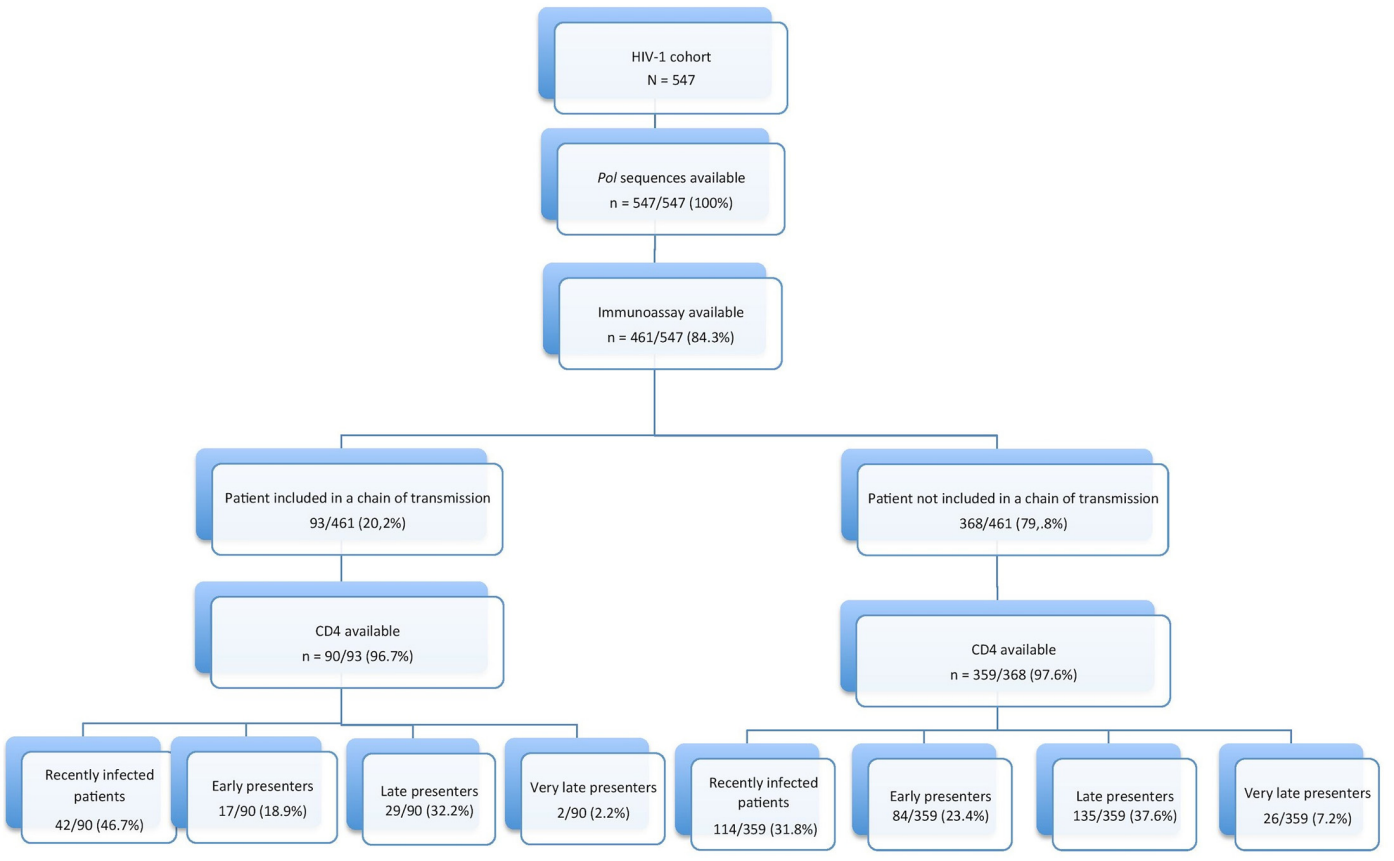
Distribution of the study population between chained and non-chained groups by time lapse between infection and genotyping. VEP: Very early presenter, EP: early presenter; LP: late presenter; and ELP: extremely late presenter.

Of the 461 patients analysed by EIA-RI, 93 (20.2%) belonged to chains. Twenty seven per-cent of VEP (42/156), 16.9% of EPs (17/101), 17.7% of LPs (29/164) and 7.1% of ELPs (2/28) were included in the clusters (Fig 2). Univariable and multivariable analysis confirmed that the recent infection defined by the EIA-RI assay as well as the age under 30 years at time of GRT were associated with belonging to a chain of infection (OR: 1.83, 95% CI: 1.14–2.93, p = 0.01 and OR: 1.74, 95% CI: 1.06–2.03, p = 0.02 respectively) (Table 2). Belonging to a pair was not associated with being recently infected or with the age at the time of GRT. Recent infection, age under 30 years at the time of diagnosis were associated with belonging to a cluster (OR: 3.65, 95 CI 1.49–8.95, p = 0.005 and OR: 2.42, 95% CI 1.05–5.85, p = 0.04 respectively) (Table 2).

**Table 2:** Factors associated with inclusion in chains, pairs and clusters.

	Population		Univariable analysis		Multivariable analysis	
	Non–chains	Chains*	Odds-Ratio	p	Odds-Ratio	p
Patients (n = 461)	368	93				
Gender (Male)	355/368	90/93	1.1 (0.31–3.93)	0.88		
Subtype B	282/368	70/93	0.92 (0.55–1.59)	0.78		
Under age 30 at the time of genotype	87/368	33/93	1.73 (1.05–2.81)	0.02	1.74 (1.06–2.03)	0.02
Recent infection (RIP)	114/368	42/93	1.83 (1.15–2.91)	0.01	1.83 (1.14–2.93)	0.01
Resistance to at least one ART	38/368	3/93	0.29 (0.08–0.96)	0.042	0.3 (0.09–1.01)	0.06
	Non-pairs	Pairs	Odds-Ratio	p	Odds-Ratio	p
Patients (n = 461)	390	71				
Gender (Male)	377/390	68/71	0.78 (0.22–2.8)	0.7		
Subtype B	301/390	51/71	0.75 (0.43–1.33)	0.33		
Under age 30 at the time of genotype	97/390	23/71	1.44 (0.84–2.5)	0.19	1.42 (0.82–2.45)	0.21
Recent infection (RIP)	128/390	28/71	1.33 (0.79–2.24)	0.28	1.32 (0.79–2.23)	0.29
Resistance to at least one ART	38/390	3/71	0.40 (0.12–1.36)	0.15	0.42 (0.13–1.40)	0.16
	Non-clusters	Clusters	Odds-Ratio	p	Odds-Ratio	p
Patients (n = 461)	439	22				
Gender (Male)	439/439	22/22	-	-		
Subtype B	333/439	19/22	2.01 (0.58–6.93)	0.27		
Under age 30 at the time of genotype	110/439	10/22	2.49 (1.04–5.90)	0.04	2.42 (1.05–5.85)	0.04
Recent infection (VEP)	142/439	14/22	3.65 (1.50–8.89)	0.004	3.65 (1.49–8.95)	0.005
Resistance to at least one ART	41/439	0/22	-	-		

HAART: Highly active antiretroviral therapy. VEP: Very Early Presenter.

*: pairs or clusters.

### Cluster characteristics

This part of the results concerned the analysis of the structure of each chain of infection. The aim was to define if there were remarkable reparation of each kind of presenters within clusters.

The mean number of patients per chain of infection was 2.2 (CI: 2–4) with most of them (82%) including only 2 patients. Subtype B was evidenced in 73% of strains involved in clustered infections.

Among chains, 59.2% contained at least one VEP, 32.7% at least one EP and 53.1% at least one LP or ELP. Chains composed by VEP only contained 8.3% of VEP (95% CI: 4.7–14.1).

**Fig 3:**
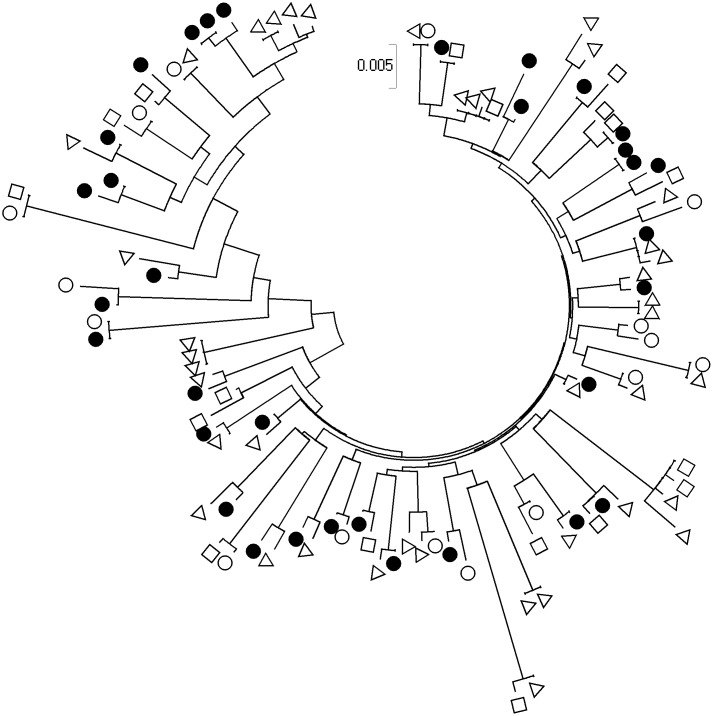
Evolutionary relationships of the 108 HIV-1 reverse transcriptase sequences involved into chained transmissions. Viruses isolated in patients infected for less than 6 months (VEP) (Δ), patients chronically-infected with CD4^+^ T-cell count >500/mm^3^ (EP) (o) or ≤500/mm^3^ (●). Subjects with unknown duration of infection (□) are shown.

In chains where all dates of infection were available by EIA-RI (n = 32), 6 (18.8%, 95% CI: 7.9–37) were composed exclusively by VEP, 9 (28.1%, 95% CI: 14.4–47) were composed only by patients considered as chronically infected presenters (EP, LP and ELP) and 17 (53.1%, 95% CI: 35–70) were composed by a mixture of both (Fig 3). These values were not different from those expected under the hypothesis that presenter’s type was randomly distributed between chains (6.46, 8.25 and 17.28 respectively) (p = 0.97). These results suggest that, VEP don’t seem to be more likely linked together than with other type of presenters or than other type of presenters.

The median time between HIV diagnosis in patients included in the same cluster was 248 days (CI: 176–320), although a shorter interval could be expected in case of frequent screening. This occurred in 30% of the clusters, where the second diagnosis was performed within three months of the first diagnosis.

## Discussion

The important results are that recently-infected patients represent one-third of individuals, diagnosis of long-standing infection remains very high, being recently infected by HIV is associated with belonging to a cluster of more than two individuals and more than 40% of VEP are included in chains of infection but do not seem to cluster preferentially together.

This study took place in a limited geographical region, spanning Parisian districts where the prevalence of HIV infected is high in MSM, with a yearly incidence as high as 3.8% cases per year, much higher than the mean figures for France and Europe [1,24,25]. These characteristics define a place where high-risk behaviour is present [26] and leads to continuing transmission in the MSM population who constitutes the largest part of the French HIV epidemic [1]. With high incidence, one would expect to find clusters as indicators of chains of transmission.

Yet, the proportion of patients involved in chains of infection (19.7%) was in the lower end compared to other reports where the proportions ranged from 30% to 70% [6,8,27,28]. This may be due to our definition of a cluster forming clade, as more restrictive definitions have been associated with fewer clusters [29,30]. A second explanation is based on the study design, as only patients who were recently diagnosed in the district could be included. The Saint-Antoine Hospital and the “Réseau Bastille” physicians’ network involved in the study provide follow-up for most individuals infected by HIV in this Paris district. The cases diagnosed elsewhere and those diagnosed before 2008 could not be taken into account here. Once again, the similar overall fraction of clustered patients than in an other study performed over a similar period using the same phylogenetic approach do not indicate that this is a major problem (19.7% vs 27.4%, p = 0.06) [29].

Besides the limitations listed above, the absence of a genetically close newly diagnosed HIV infected individual may indicate that a substantial fraction of transmitters are either undiagnosed or diagnosed but untreated. In France, approximately 20% of infected individuals are undiagnosed [31], but most diagnosed cases are involved in HIV care that should limit transmission. These results also draw attention to the majority of non-chained individuals. Their sexual behaviour, their use of screening test for HIV and their partner characteristics may be central to the epidemic spread [32]. This puts the focus on the undiagnosed cases in this population, all the more that a significant fraction of newly diagnosed cases had a longstanding infection.

VEP were more likely to be part of a chain. VEP did not cluster together more than chance predicted with only 18.8% of the chains involving only VEP. The proportion VEP included in chains built only by VEP is similar to that reported in previous studies based on acutely-infected patients in France using the same phylogenetic approach (8.3%, (95% CI: 4.7–14.1) vs 12.7%, (95% CI: 10.7–14.9), p = 0.16) (30).

Recent studies have suggested that newly diagnosed HIV-positive patients should cluster predominantly with other recently diagnosed HIV-positive [6,8,12,27–29,32,33] and indicate that transmission occurs early after infection. Debate continues about the possible predominant role of recently infected patients in the dynamics of the HIV epidemics. Most of the studies linking viral phylogenetics to time of infection and its relevance in the spread of HIV conclude that acutely infected patients are the primary drivers of the epidemic [6,8,10,11]. This is supported by the large proportion of VEP involved into phylogenetic chains predominantly of more than 2 individuals. However, the observed distribution of VEP into chains can also suggest that new infections are not predominantly due to acute transmission, as already suggested [29]. These discordant results could be in favour of the presence of different processes of transmission that can be distinguished by the fact that they produce pairs or clusters and that involve more predominantly individuals at a particular stage of their infection. Furthermore, it is important to say that with these kinds of data, stage of infection of the source is unknown at the time of transmission. Thus, it is hard to demonstrate that recently infected individuals are contributing to onward infection because the chronically infected individuals they are clustered with could be as likely to have been the source for their infection as to have been infected by them. Recent model studies estimate that near a half of transmission occur within the first year of infection in the HIV epidemic among MSM [34]. More precise estimations are needed to evaluate the impact of prevention strategies such as TaSP. This is a major concern, which needs further modelling studies based on these types of data and exploring possible onward transmission within clusters with regard to estimated stage of infection of each possible sources.

Finally, this study is one among the very few which involved a genotypic approach combined with a non-subjective method to define recently infected patients [30, 33]. The EIA-RI assay has already proven its efficacy for the National HIV case surveillance in France [22,24,35]. Definition of acute or recent infection is a major concern and might explain variations in results among studies on the same subject [12]. A biological methodology such as the one we used to identify recent infections seems to be more reproducible than declaration or biological indicators such as CD4 T-cell count, seroconversion, p24 antigen assay or frequency of ambiguous nucleotides in sequencing to date the infection [34–37].

## Conclusion

Our study confirmed that being recently diagnosed is associated to belonging to a cluster of more than two individuals. Chains of infection involves a large part of recently infected individuals but they do not cluster together more than chance predicted, The proportion of patient infected for more than 6 month at the time of diagnosis remains high and they represent the majority of clustered individuals. A large percentage of our population is not included in chains, suggesting that most onward transmission could be due to an undiagnosed or untreated patient. These results are of particular importance to the development of prevention strategies such as ‘treatment as prevention’ or ‘pre-exposure prophylaxis’ in this population engaged in high risk behaviours.
